# Heat Transfer and Flow Structures of Laminar Confined Slot Impingement Jet with Power-Law Non-Newtonian Fluid

**DOI:** 10.3390/e20100800

**Published:** 2018-10-18

**Authors:** Yan Qiang, Liejiang Wei, Xiaomei Luo, Hongchao Jian, Wenan Wang, Fenfen Li

**Affiliations:** 1 College of Energy and Power Engineering, Lanzhou University of Technology, Lanzhou 730050, China; 2 Key Laboratory of Fluid Machinery and System, Lanzhou 730050, China; 3 China North Vehicle Research Institute, Beijing 100071, China

**Keywords:** laminar impinging slot jet, power-law index, consistency index, Power-Law Non-Newtonian fluid

## Abstract

Heat transfer performances and flow structures of laminar impinging slot jets with power-law non-Newtonian fluids and corresponding typical industrial fluids (Carboxyl Methyl Cellulose (CMC) solutions and Xanthangum (XG) solutions) have been studied in this work. Investigations are performed for Reynolds number *Re* less than 200, power-law index *n* ranging from 0.5 to 1.5 and consistency index *K* varying from 0.001 to 0.5 to explore heat transfer and flow structure of shear-thinning fluid and shear-thickening fluid. Results indicate that with the increase of *n*, *K* for a given *Re,* wall Nusselt number increases mainly attributing to the increase of inlet velocity *U*. For a given inlet velocity, wall Nusselt number decreases with the increase of *n* and *K*, which mainly attributes to the increase of apparent viscosity and the reduction of momentum diffusion. For the same *Re*, *U* and *Pr*, wall Nusselt number decreases with the increase of *n.* Among the study of industrial power-law shear-thinning fluid, CMC solution with 100 ppm shows the best heat transfer performance at a given velocity. Moreover, new correlation of Nusselt number about industrial fluid is proposed. In general, for the heat transfer of laminar confined impinging jet, it is best to use the working fluid with low viscosity.

## 1. Introduction

Heat transfer enhancement technologies have drawn much more attentions in recent years for the intense demand in the industrial fields. Generally, the machining of rough or extended surfaces and vortex generators, as well as the promotion of working fluid properties is considered as the passive methods. While the active methods such as forced channel flow, impinging jets, mechanical vibration and electromagnetic field consume extra energy.

Among these heat transfer enhancement methods, impinging jets have the highest known single phase local heat transfer rate but low drop pressure, which is attractive for electronic thermal management [1]. Basic parameters such as Reynolds number and jet-to-surface spacing significantly influence flow fields and heat transfer performances [2,3,4]. What’s more, the further heat transfer enhancement of impinging jets can be obtained by combining other active or passive methods, which has been widely investigated by many scholars [5,6,7,8]. For example, single jet/jet arrays impinging onto dimpled target surface are numerically investigated [9]. The associated results show that the average Nusselt number increases with the variations of dimple relative depth. Selimefendigil et al. [10] studied the effects of pulsating frequency, Reynolds number and nanoparticle volume fraction on the fluid flow and heat transfer characteristics. Shojaeizadeh et al. [11] analyzed the bed roughness effects on the variations of turbulent confined wall jets. Geng et al. [12] conducted a series of experiments about unsteady impinging jets with different waveforms. Yarmand et al. [13] analyzed the entropy generation in the square cross section tube, which had a constant heat flux under turbulent flow and nanofluids condition. Li et al. [14] numerically studied single jet impinges on dimpled surface using Al_2_O_3_-Water nanofluids. They found that the average Nusselt number increases with the variations of nanoparticle volume concentration (*φ*). From the previous literatures, it can be concluded that combinations of several heat transfer enhancement technologies have become one heated topic. Impinging jets with unconventional working fluid seems to be potential for strengthening heat transfer.

Non-Newtonian fluid has drawn some attentions in recent years for its obvious advantages of drag reduction and heat transfer enhancement in channel flow [15,16,17,18]. So, via Non-Newtonian fluid, impinging jet has been studied. For example, Maleki et al. [19,20] investigated the characteristics of the flow and heat transfer of non-Newtonian nanofluids in the porous surface and they also analyzed the features of viscous dissipation and heat absorption and generation under heat transfer and fluid flow of pseudo-plastic nanofluid condition. Poh et al. [21] investigated a single axis-symmetric semi-confined laminar impinging jet with CMC (carboxymethyl cellulose) solutions. Their results showed that for a fixed generalized Reynolds number, when power-law index decreases, the increase of inlet velocity leads to the growing of Nusselt number. For a fixed inlet velocity, Nusselt numbers remain unchanged regardless of the changes of Reynolds number and CMC concentration. Hassan et al. [22] deeply discussed the generation of entropy in the flowing Nanofluid fluid which was employed in the micro and minichannels. Srisamran et al. [23] numerically simulated the flow and mix behavior of two 2D steady confined laminar impinging jets using shear-thinning fluid CMC solutions. They found that jet interaction in the impingement zone increased and the size of recirculating bubbles enlarged with the increase of *Re*. Hooshmand et al. [24] numerically investigated the effects of magnetic fields on the variations of heat transfer and generation of entropy over an axisymmetric stretching plate structure. Zhao et al. [25] theoretically examined the spread of shear-thinning and shear-thickening liquid jet over a horizontal plate, which indicated that highly viscous and strongly shear-thinning fluids difficultly spread, even compared with highly viscous and simultaneously highly shear-thickening fluids, which results from the interaction between the shear-thinning character and the overall viscosity of the fluid. Abbas et al. [26] discussed the generation of entropy of the peristaltic nanofluids which flowed in the channels, having the compliant walls. Yilbas et al. [27] studied the entropy characteristics of the Non-Newtonian fluid which flowed in one annular pipe under constant viscosity condition. Shojaeizadeh et al. [28] employed finite volume method to investigate the laminar mixed convection heat transfer of power-law non-Newtonian fluids in square enclosures. Gharraei et al. [29] numerically studied the non-Newtonian multiple impinging jets at the Reynolds number 100–200, power-law index 0.4–1.6 and dimensionless jet-to-plate spacing 0.25–1.0. They found that under the same Reynolds number and consistency coefficient condition, with the increase of power-law index, wall Nusselt number increased because of the increasing of inlet velocity. Yousefi-Lafouraki et al. [30] investigated entropy generation with non-Newtonian nanofluid of a confined laminar slot impinging jet. The working fluid is a CMC solution with TiO_2_ nanoparticles, exhibiting pseudoplastic behavior. Overall, non-Newtonian fluid whose viscosity is sensitive to shear strain rate may be effective on impinging jets with manifest shear flow.

In the review of published literatures, the combination studies of impinging jets and non-Newtonian fluid are necessary for heat transfer enhancement and the researches especially for the studies of the heat transfer characters on a confined impinging slot jet by employing power-law liquid are relatively few. More work is still needed to obtain better heat transfer performance. In this study, a confined impinging slot jet was numerically investigated considering the effects of power-law index *n*, consistency index *K* and inlet velocity *u*. Moreover, industrial-fluid solutions such as CMC and xanthane are also examined. By this study, several variation tendencies of Nusselt number with diverse physical parameters can be obtained so that the desirable working fluid can be found.

## 2. Numerical Methods and Validation

### 2.1. Governing Equations

In the study, non-Newtonian fluid which behaves as temperature is independent of power-law fluid was selected as the working fluid. Governing equations of continuity, momentum and energy of the steady, laminar and incompressible flow in a Cartesian reference frame are given as follows [31].

(1) $$\frac{\partial u}{\partial x}+\frac{\partial v}{\partial y}=0$$

(2) $$\rho \left(u\frac{\partial u}{\partial x}+v\frac{\partial u}{\partial y}\right)=\frac{\partial \tau_{ii}}{\partial x}+\frac{\partial \tau_{ji}}{\partial y}$$

(3) $$\rho \left(u\frac{\partial u}{\partial x}+v\frac{\partial u}{\partial y}\right)=\frac{\partial \tau_{ji}}{\partial x}+\frac{\partial \tau_{jj}}{\partial y}$$

(4) $$\rho c_{p}\left(u\frac{\partial T}{\partial x}+v\frac{\partial T}{\partial y}\right)=\lambda \left(\frac{\partial^{2}T}{\partial x^{2}}+\frac{\partial^{2}T}{\partial y^{2}}\right)$$

where *τ_ij_* is the viscous stress tensor and subscripts *i* and *j* represent the normal direction of action surface and direction of the stress component on the aforementioned surface, respectively. *T* is the temperature. *u* and *v* are the velocity. *x* and *y* are the Cartesian coordinates. *ρ*, *c_p_* and *λ* are the fluid density, specific heat and thermal conductivity, separately. Moreover, the pressure terms are additionally included in the normal stress tensor *τ_ii_*_._

### 2.2. Physical Model and Boundary Conditions

**Figure 1:**
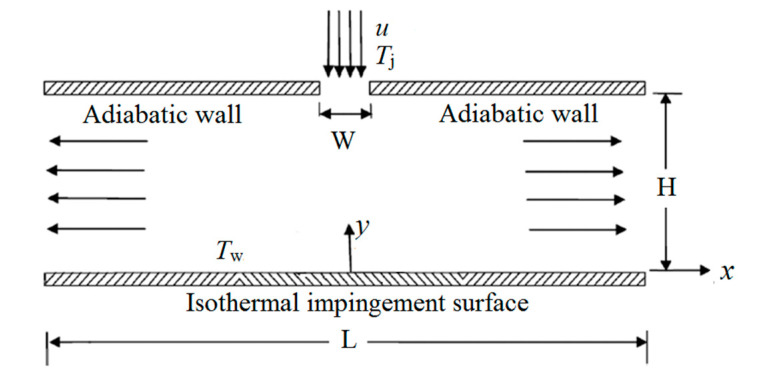
Physical model of a two-dimensional confined impinging jet.

The physical model of the confined slot impinging jet is similar to that of [32]. As seen in Figure 1, the jet impinges from the jet exit of width *W =* 0.0062 mm. Uniform velocity *U* ranges from 0.02 m/s to 0.1 m/s and the uniform temperature is *T_j_ =* 293 K. Normally, the isothermal target surface temperature is *T*_wall_ = 313 K. *H* represents the distance between jet exit and the target surface. *L* is the plate length, respectively. In our study, the *H*/*W* is fixed to be 4 and *L*/*W* is fixed to be 100.

The numerical simulation was conducted with Fluent. A second-order upwind scheme and the SIMPLE algorithm are employed to simulate the steady-state laminar flow. Material properties of the working fluid are created by using the non-Newtonian power-law fluid model. The minimum and the maximum viscosity are set at 0 Pa·s*^n^* and 1000 Pa·s*^n^*, respectively. Various power-law liquids can be obtained by changing power-law index *n*, ranging from 0.5 to 1.5 and consistency index *K*, ranging from 0.001 to 0.5. The fluid density, specific heat and thermal conductivity are assumed to be constant under *T_j_* = 293 K condition in Table 1. All of the other surfaces are adiabatic and non-slip walls. At outlet, the pressure is set. The residuals of Nusselt number on the stagnation point and average velocity of the whole computational domain, as well as the default quantities, such as continuity, velocities and energy, are monitored to judge the convergence of simulation, in which the convergence criteria are set as 10^−6^.

### 2.3. Data Reduction

The apparent temperature independent dynamic viscosity η of the power-law fluid is given by

(5) $$\eta =K\gamma^{n-1}$$

Here, *K* is the consistency index; *n* is the power-law index which determines the class of the fluid; *γ* is the shear strain rate. As *n =* 1, the fluid is Newtonian fluid; as *n* < 1, the fluid viscosity decreases with the increase shear strain rate, exhibiting pseudo-plastics (shear-thinning) behavior; as *n* > 1, the fluid whose viscosity increases with the increase shear strain rate is the dilatant (shear-thickening) fluid.

Furthermore, the Reynolds number and Prandtl number [17] are defined as:

(6) Re=ρWnU2−nK

(7) $$\mathrm{Pr}=\frac{c_{p}K}{\lambda }{\left(\frac{U}{W}\right)}^{n-1}$$

where *U* is the inlet velocity, *W* is the jet exit width, *ρ*, *c_p_* and *λ* denote the fluid density, specific heat and thermal conductivity, respectively.

The heat transfer performance is characterized by Nusselt numbers, expressed as

(8) $$Nu=\frac{Wh}{\lambda }$$

Here, *h* is the heat transfer coefficient which is defined as

(9) $$h=\frac{q}{T_{w}-T_{j}}$$

where *q* is the heat flux, *T_w_* is the temperature of the target surface and *T_j_* represents the temperature of the jet.

Pumping power [27] is defined as:

(10) $$PP=Q\Delta P_{t}$$

Here, *Q* is the volume flow rate, ∆*P_t_* is the difference of total pressure between the inlet and outlet cross-section.

### 2.4. Grid-Independence Check

**Figure 2:**
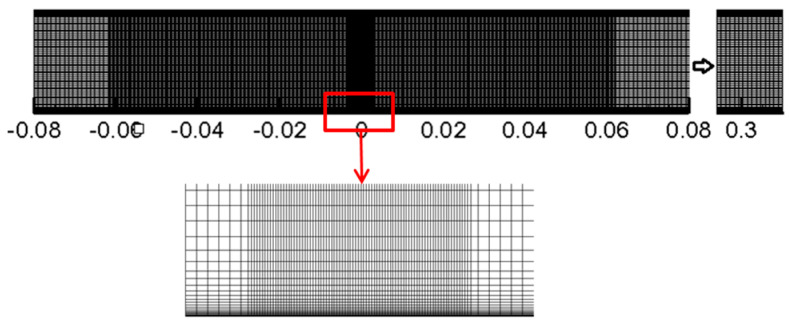
Computational grid for solution domain.

The grid independence check is carried out by testing four different grids under *U =* 0.016 m/s, *Re =* 100, *H*/*W =* 4, *n =* 1, *K =* 9.93 × 10^−4^ Pa·s. As seen in Table 2, total grid nodes were 30,744 (549 × 56), 60, 138 (771 × 78), 82,440 (916 × 90) and 123,424 (1102 × 112), respectively. When the mesh changes from grid 3 to grid 4, the deviation of the average Nusselt number *Nu*_ave_ of the whole plate is 0.054%, which is small enough. To guarantee the accuracy of results and reduce the computational resource cost, grid 3 is chosen for the following study. The grid diagram and magnification of refined grids near the impingement region and the walls are shown in Figure 2.

### 2.5. Method Validation

**Figure 3:**
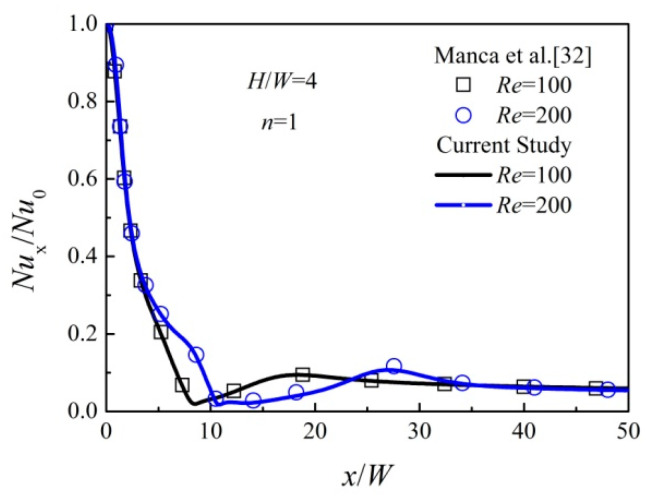
Validation of results with data.

The impinging jet using special non-Newtonian liquid pure water as the working fluid is employed to validate the simulation results with the available data given in [32]. The same physical model and boundary conditions are adopted for the cases with the pure water of *n =* 1, *K =* 9.93 × 10^−4^ Pa·s, inlet velocity ranging from 0.016 m/s to 0.032 m/s, *Re* ranging from 100 to 200 and *H*/*W =* 4. As observed in Figure 3, the *Nu*_x_/*Nu*_0_ distributions show a good agreement with that of the literature [32] for all the validated cases, in which *Nu*_x_ and *Nu*_0_ represent local Nusselt number and Nusselt number at the stagnation point, respectively. Therefore, the numerical method simulating the power-law liquid impinging jet can be employed in the following investigations.

## 3. Results and Discussions

### 3.1. Effects of n and K Based under a Given Re

**Figure 4:**
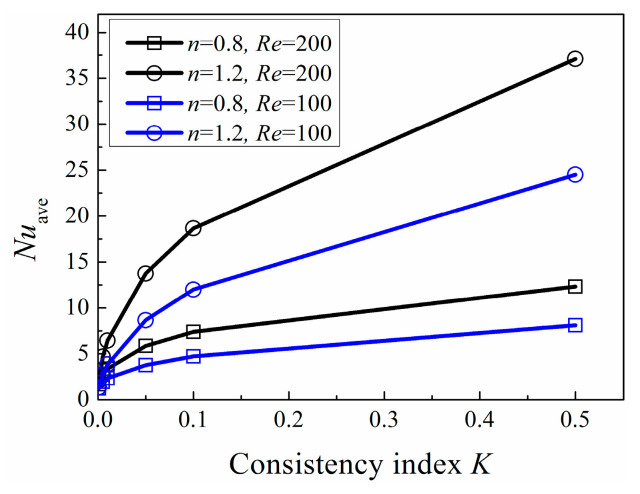
Variation of Area-averaged Nusselt number at various *Re***.**

**Figure 5:**
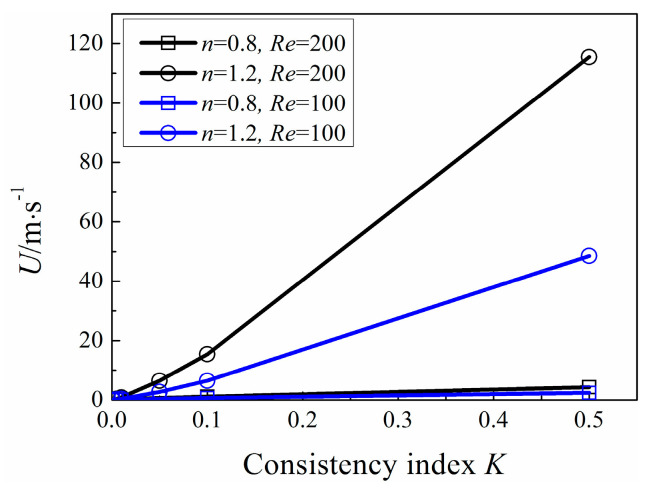
Variation of inlet velocity *U* with *K.*

In order to simulate extensive fluid in nature, *K* ranges from 0.001 to 0.5, which varies in a large scale in order to simulate extensive fluid in nature, the associated results show large variable ranges. As shown in Figure 4, *Nu*_ave_ is influenced by Reynolds number *Re*, power-law index *n* and consistency index *K*, simultaneously. *Nu*_ave_ increases with *Re*. For a fixed Reynolds number, *Nu*_ave_ increases with the increase of power-law index *n* and consistency index *K*, respectively. Figure 5 gives the corresponding inlet velocity *U* calculated via Equation (6). It can be seen that the conditions with high *Nu*_ave_ tend to get high inlet velocity. The increase of *Nu*_ave_ mainly attribute to the increase of inlet velocity *U.*

**Figure 6:**
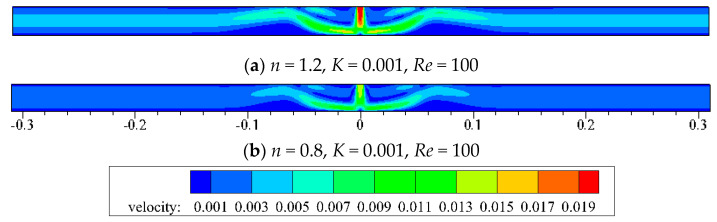
Velocity contour for *Re =* 100.

**Figure 7:**
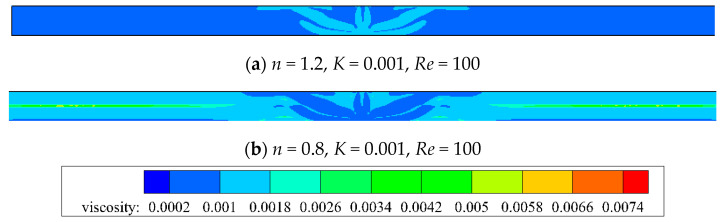
Apparent viscosity contour for *Re =* 100.

From Figure 6, it is observed that the impact momentum of *n =* 1.2 is larger than that of *n =* 0.8 for a fixed *Re =* 100. Figure 7 also shows the corresponding apparent viscosity contour, which has closed relation with shear strain rate. When jet impinges from the exit, the shear-layer between the jet and the ambient fluid grows along the flow direction, which leads to high shear strain rate because of high velocity gradient. The potential core (center part of the jet) where the velocity gradient is low maintains an almost constant velocity. As the shear layer spreads to the center of the jet, the associated potential core vanishes. When jet impinges on the target wall, it turns in the transverse direction and the flow is along the wall. As *x*/*W* is about equal to 7, the wall jet turns its direction upward because of the vortex which is near the impinged wall. As *x*/*W* is larger than 25, the shear flow runs parallel and the flow is out of the domain exit. So, for shear-thinning fluid (*n =* 0.8), the apparent viscosity of shear-layer is lower than that of the potential core. The apparent viscosity is largely away from the center of the domain, while the shear-thickening fluid (*n =* 1.2) shows the opposite distribution.

**Figure 8:**
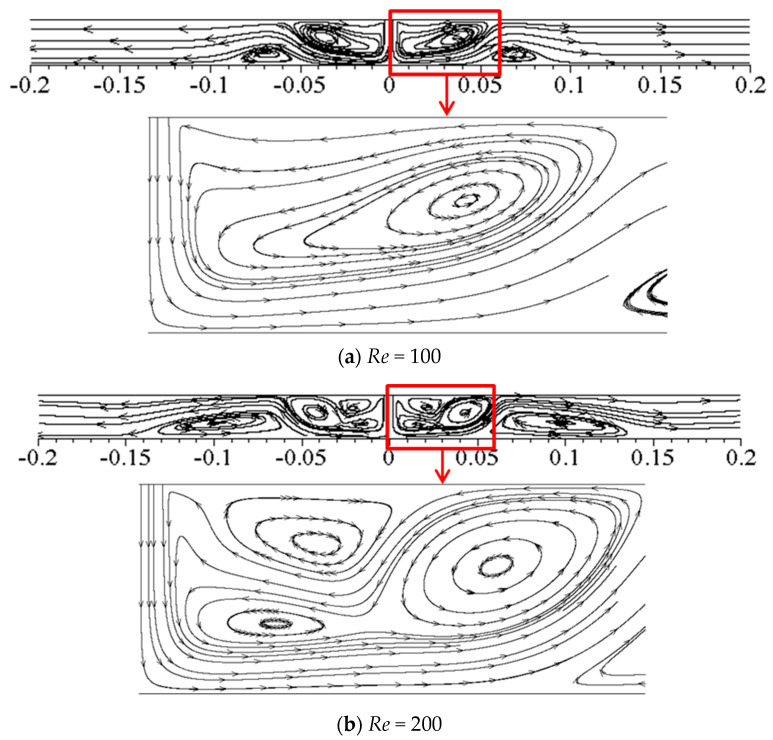
Streamlines for *Re =* 100 and 200.

As shown in Figure 8, the flow is steady and symmetric. When *Re =* 100, the formation of the main re-circulating vortex near the jet inlet and secondary vortices on the impinged wall can be observed. Compared with *Re =* 100, the flow structure of *Re =* 200 changes significantly. The main re-circulating vortex is split into two main cores and a new vortex is generated near the jet inlet. Secondary vortex on the lower wall is stretched to the lateral exit along the lower wall. When *Re* increases further, the flow structure becomes more complex so that the flow becomes unsteady and unsymmetrical with complicated vortices.

What’s more, the differences of total pressure between the inlet and outlet cross-section is in a wide range, which mainly are affected by inlet velocity *U*. Therefore, it is better to compare the different working fluid based on the same inlet velocity if the pumping power is considered.

### 3.2. Effects of n and K Based on the Same Inlet Velocity U

**Figure 9:**
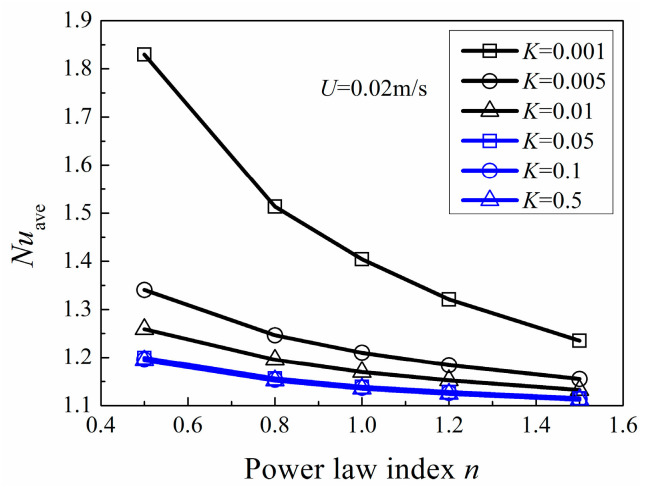
Area-averaged Nusselt number with *n*.

Figure 9 indicates the variation of *Nu*_ave_ with power-law index *n* for various consistency index *K* at the same inlet velocity *U*. It can be seen that *N*u_ave_ decreases with an increase of power-law index *n* and *K*, respectively. When *K* is larger than 0.05, the effects on *Nu*_ave_ for different *K* are insignificant, which mainly attribute to the small differences of *Re*.

**Figure 10:**
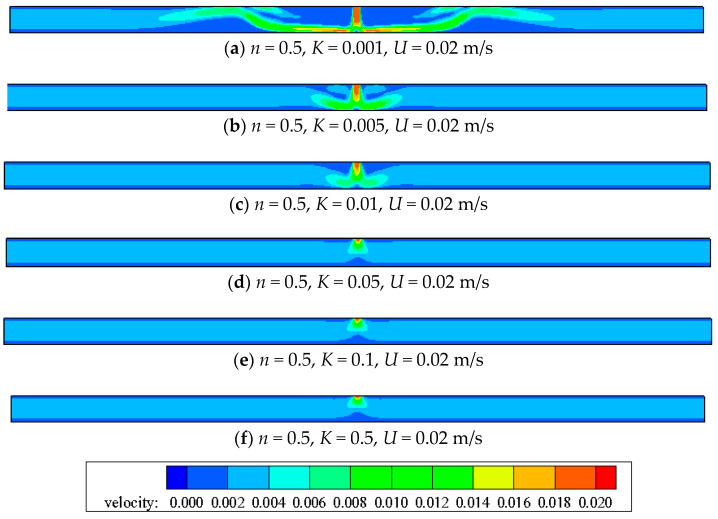
Velocity contour with the same inlet velocity when *n =* 0.5.

**Figure 11:**
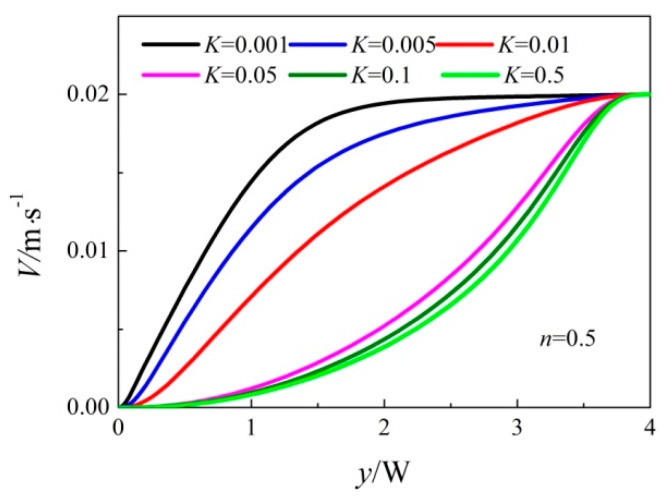
Center velocity distribution with the same inlet velocity when *n =* 0.5.

The effects of *n* and *K* on jet flow are manifested mainly by influencing the apparent viscosity. From Equation (5), the apparent viscosity *η* increases with *n* under the same *K* condition. When *n* is identical, the apparent viscosity *η* increases with *K*. As seen in Figure 10 and Figure 11, despite of the same inlet velocity, the jet momentum diffusion becomes weaker with the increase of *K* for the increasing apparent viscosity. Furthermore, the center velocity decays quickly with the increase of *K*, which is not good for the heat transfer.

**Figure 12:**
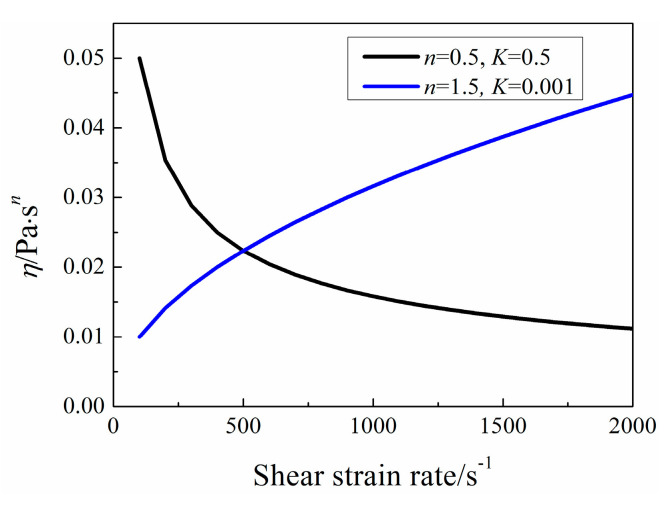
Variation of apparent viscosity *η* with shear strain rate.

When *n* < 1 with large *K* such as *n =* 0.5 and *K =* 0.5, the heat transfer performance is not better than that when *n* > 1 with small *K* such as *n =* 1.5 and *K =* 0.001, which is connected with the apparent viscosity. Figure 12 clearly states that the variation of apparent viscosity *η* with shear strain rate for a specific case. When *U =* 0.02 m/s, the calculated maximum shear strain rate is about 110 s^−1^. Under this condition, shear-thickening fluid is better using as heat transfer working fluid due to the shear strain rate is less than 500 s^−1^. So, when the pumping power is fixed, it is better to adopt fluid with small apparent viscosity for good heat transfer enhancement.

**Figure 13:**
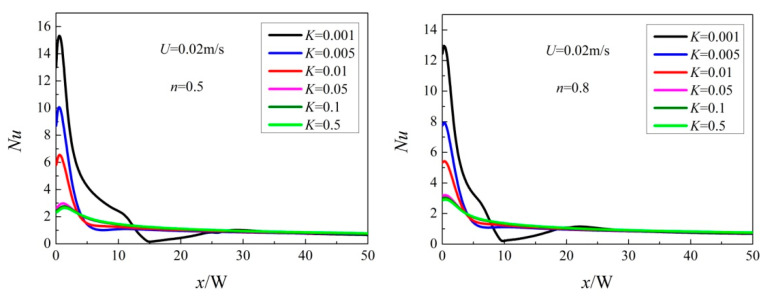

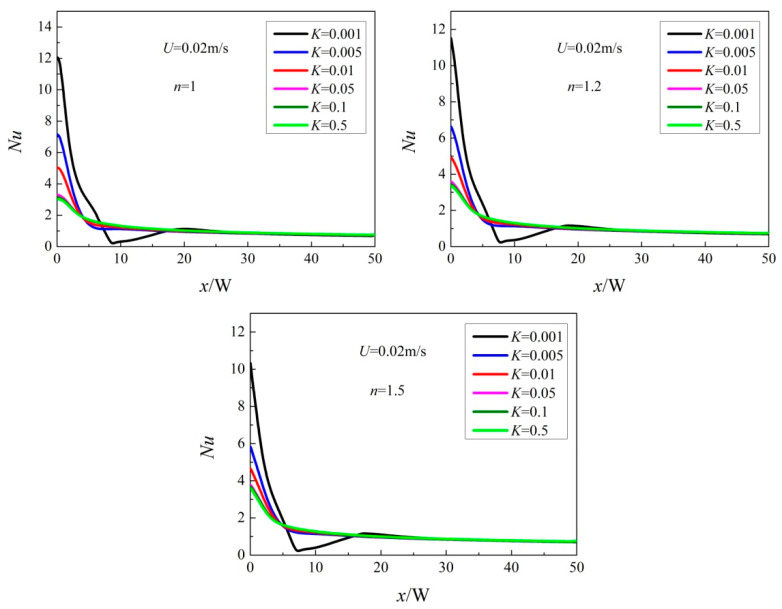
Local Nusselt number at various *n.*

Figure 13 reveals the local Nusselt number distribution for diverse power-law index *n*. When *n* < 1, the local Nusselt number does not attain the peak at the stagnation point. What’s more, with the decrease of *n*, the deviation between the stagnation Nusselt number and the maximum value increases. When *n* is not less than 1, the local Nusselt number reaches the maximum at the stagnation point.

### 3.3. Effects of n and K Based on the Same Re, Pr and U

According to the definition of *Pr*, *K* can be defined as:

(11) $$\frac{1}{K}=\frac{c_{p}}{\lambda \mathrm{Pr}}{\left(\frac{U}{W}\right)}^{n-1}$$

Then, putting Equation (11) into Equation (6), we can get it as follows:

(12) Re=ρWnU2−nK=cpλPr(UW)n−1·ρWnU2−n=ρcpλPrWU

**Figure 14:**
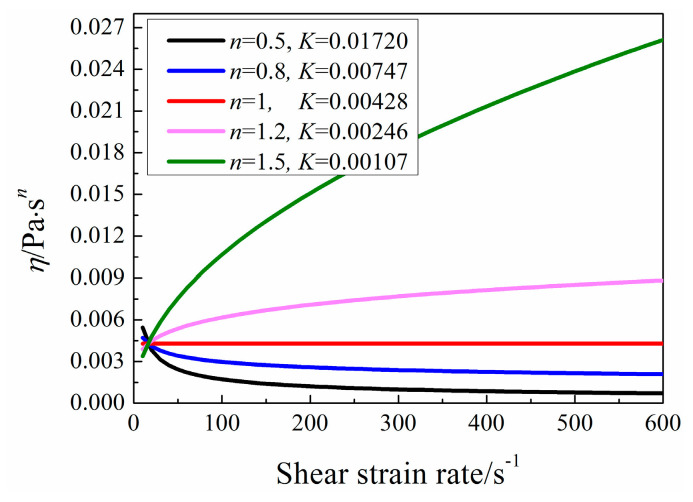
Variation of apparent viscosity *η* with different shear strain rate.

So, when *Pr* and *U* are the constants, the same *Re* is obtained. By changing *n* and *K* which are based on the same *Pr*, different fictitious power-law fluid could be got, as displayed in Table 3. Under such conditions, results are singly influenced by the sole factor *n*. Furthermore, variations of apparent viscosity of five fictitious fluids are exhibited in Figure 14. With the increase of *n*, the apparent viscosity increases on the whole.

**Figure 15:**
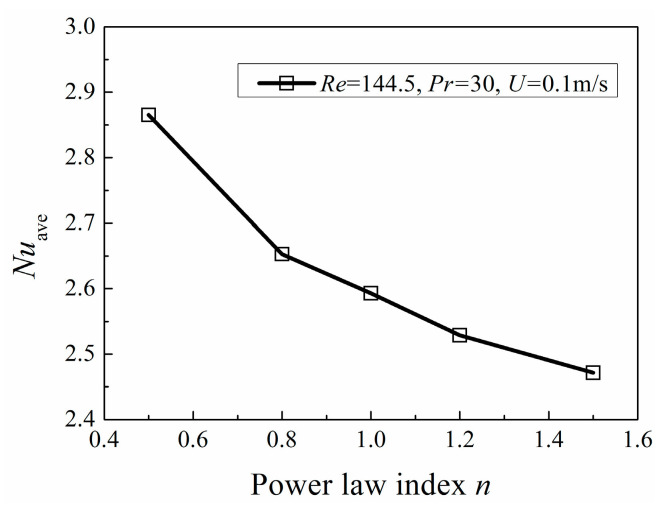
Area-averaged Nusselt number with *n* under the same *Re* and *Pr.*

Figure 15 indicates that the area-averaged Nusselt number decreases with the increase of *n* under the condition of *Re =* 144.5, *Pr =* 30 and *U =* 0.1 m/s. With the identical *U*, *Re* and *Pr*, fluid with low viscosity tends to have better heat transfer performance.

### 3.4. Typical Industrial Fluids Study

The polymer aqueous solutions of CMC and XG represent shear-thinning behavior, which are employed in this study. Their physical properties except the dynamic viscosity are considered as the same as that of water at 293 K for low temperature difference and concentration. The dynamic viscosity changes with shear strain rate according to the rule of the power-law exponent. The power-law index *n* and consistency index *K* for different concentrations is listed in Table 4.

**Figure 16:**
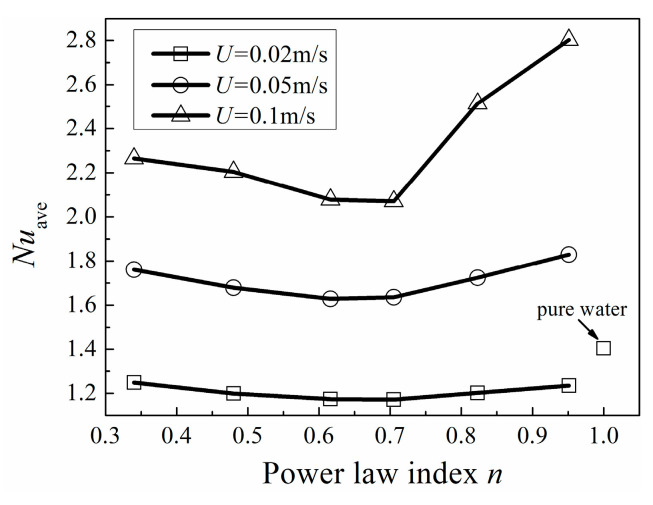
Area-averaged Nusselt number with different *n* at various *U.*

**Figure 17:**
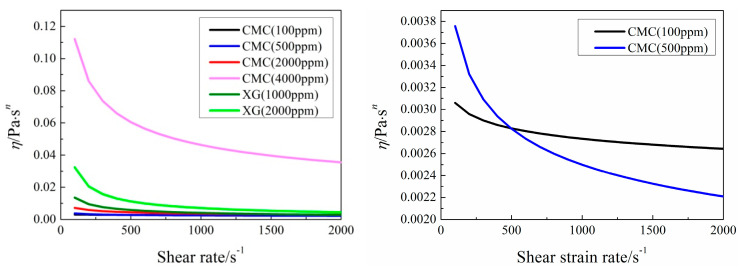
Variation of apparent viscosity *η* with shear strain rate.

It can be seen from Figure 16, *Nu*_ave_ decreases and then increases with the increase of *n* under various *U* conditions. Because when *U* is larger than 0.05 m/s, the *Re* of pure water is larger than 309, the flow is unsteady, which is beyond our study. So, the industrial shear-thinning fluids are compared with pure water only when *U =* 0.02 m/s. Pure water has better heat transfer performance which mainly attributes to its low viscosity. As seen in Figure 17, the least apparent viscosity of industrial shear-thinning fluid under shear strain rate (about 110 s^−1^) in this study is about 0.003 Pa·s*^n^*, which is larger than that of pure water of 0.000993 Pa·s*^n^*. Moreover, CMC solution with 100 ppm concentration shows the best heat transfer performance among the industrial shear-thinning fluids in this study.

### 3.5. Correlation

Correlation of *Nu*_ave_ is given based on the industrial fluids results obtained in the present work, because the given fictitious fluid is in a wide range which is difficult to fit a correlation. The correlation formula with five undetermined coefficients is expressed as [16]:

(13) Nuave=A⋅PrB⋅ReC⋅(3n+14n)D⋅En

Table 5 lists the coefficients of *A*, *B*, *C*, *D*, *E* and RMSE (Root Mean Square Error).

**Figure 18:**
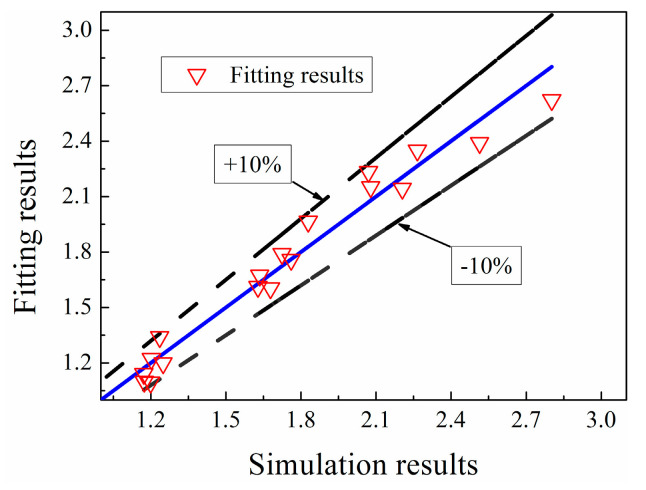
Comparison of simulation results and fitting results.

The simulation results are compared with fitting results. Their maximum deviation is 8.7%, as displayed in Figure 18. Therefore, the correlation could be further used for the prediction of heat transfer performance of industrial fluids in the study range due to the good fit.

## 4. Conclusions

Heat transfer of fictitious and industrial power-law non-Newtonian fluid in laminar confined impinging jet is investigated. The main conclusions obtained are as follows.

(1) For a given Reynolds number, the average Nusselt number increases with *n* and *K*, respectively, which mainly attributes to the increase of inlet velocity. With the increase of *Re*, the average Nusselt number increases and the flow becomes complex with more vortices.
(2) For the given Reynolds number, Prandtl number and inlet velocity, the average Nusselt number decreases with the increase of *n*.
(3) Industrial shear-thinning fluids are investigated, which is not better than pure water at the given inlet velocity due to the high viscosity under the shear strain rate in this study. Among these shear-thinning fluids studied in this paper, CMC solution with 100 ppm concentration shows the best heat transfer performance. What’s more, a correlation of *Nu*_ave_ is proposed.
(4) For laminar confined impinging jet at a given inlet velocity, application of non-Newtonian fluid has no significant influence on heat transfer enhancement. To obtain good heat transfer enhancement, fluid with low viscosity is the reasonable choice. Moreover, turbulent impinging jets with non-Newtonian fluid need more future research.

## Figures and Tables

**Table 1:** Properties of water at 293 K.

Material	*ρ*/kg·m^−3^	*c_p_*/J·kg^−1^·K^−1^	*μ*/Pa·s	*λ*/W·m^−1^·K^−1^
water	998.2	4182	9.93 × 10^−4^	0.597

**Table 2:** Grid Independence Check.

Grid	Nodes	*Nu*_ave_	Deviation between Adjacent Grid %
1	30,744	1.2484	0.109
2	60,138	1.2498	0.124
3	82,440	1.2513	0.054
4	123,424	1.2520	/

**Table 3:** Power-law and corresponding consistency indexes of fictitious fluid for *Re* = 144.5, *Pr* = 30 and *U =* 0.1 m/s.

*n*	*K*/kg⋅m^−1^⋅s^*n*^^−2^
0.5	0.01720
0.8	0.00747
1	0.00428
1.2	0.00246
1.5	0.00107

**Table 4:** Power-law and consistency indexes for the solutions of carboxyl methyl cellulose (CMC) [22] and XG [33].

*n*	*K*/kg⋅m^−1^⋅s^*n*^^−2^	Polymer Aqueous Solutions
0.9512	0.00383	CMC (100 ppm)
0.8229	0.00849	CMC (500 ppm)
0.7051	0.02792	CMC (2000 ppm)
0.6161	0.6572	CMC (4000 ppm)
0.48	0.149	XG (1000 ppm)
0.34	0.678	XG (2000 ppm)

**Table 5:** Coefficients of the correlation.

	A	B	C	D	E	RSME
*Nu*_ave_	0.0240	0.4153	0.4168	1.7190	3.4855	0.0017

## Nomenclature

CMC	Carboxyl Methyl Cellulose
*c_p_*	special heat of the fluid (J·kg^−1^·K^−1^)
*h*	heat transfer coefficient (W m^−2^·K^−1^)
*H*	height of nozzle-to-plate distance (m)
*K*	consistency index of power-law fluid (kg·m^−1^·s*^n^*^−2^)
*L*	length of the target plate (m)
*Nu*	Nusselt number based on nozzle diameter
*n*	power-law index
*Pr*	Prandtl number
Δ*P_t_*	total pressure difference between the inlet and outlet sections
*Q*	volume flow rate
*q*	wall heat flux (W·m^−2^)
*Re*	Reynolds number based on nozzle width
*T*	temperature (K)
*u*	velocity of the fluid in the *x* direction (m·s^−1^)
*v*	velocity of the fluid in the *y* direction (m·s^−1^)
*U*	inlet uniform velocity (m·s^−1^)
*W*	nozzle width (m)
*x*	coordinate parallel to plate (m)
XG	xanthangum
*y*	coordinate normal to plate (m)
**Greek symbols**	
*η*	apparent dynamic viscosity of the power-law fluid (kg·m^−1^·s*^n^*^−1^)
*λ*	thermal conductivity of the fluid (W·m^−1^·K^−1^)
$\dot{\gamma }$	shear strain rate (s^−1^)
*ρ*	density of the fluid(kg·m^−3^)
*τ_ij_*	stress tensor components
*j*	jet temperature
*w*	wall temperature
**Subscripts/superscripts**	
ave	area-average
